# Diffusive and martensitic nucleation kinetics in solid-solid transitions of colloidal crystals

**DOI:** 10.1038/ncomms14978

**Published:** 2017-05-15

**Authors:** Yi Peng, Wei Li, Feng Wang, Tim Still, Arjun G. Yodh, Yilong Han

**Affiliations:** 1 Department of Physics, Hong Kong University of Science and Technology, Clear Water Bay, Hong Kong SAR, China; 2 Department of Physics and Astronomy, University of Pennsylvania, Philadelphia, Pennsylvania 19104, USA; 3 The HKUST Shenzhen Research Institute, Shenzhen 518057, China

## Abstract

Solid–solid transitions between crystals follow diffusive nucleation, or various diffusionless transitions, but these kinetics are difficult to predict and observe. Here we observed the rich kinetics of transitions from square lattices to triangular lattices in tunable colloidal thin films with single-particle dynamics by video microscopy. Applying a small pressure gradient in defect-free regions or near dislocations markedly transform the diffusive nucleation with an intermediate-stage liquid into a martensitic generation and oscillation of dislocation pairs followed by a diffusive nucleus growth. This transformation is neither purely diffusive nor purely martensitic as conventionally assumed but a combination thereof, and thus presents new challenges to both theory and the empirical criterion of martensitic transformations. We studied how pressure, density, grain boundary, triple junction and interface coherency affect the nucleus growth, shape and kinetic pathways. These novel microscopic kinetics cast new light on control solid–solid transitions and microstructural evolutions in polycrystals.

## Abstract (web summary)

Solid-solid transitions between different crystalline structures have broad implications in earth science, steel and ceramic materials. Peng *et al*. show a transformation pathway that starts off as being martensitic then switches to diffusive at the single particle level in a colloidal system under pressure.

Solid–solid (s–s) transitions are arguably the most common type of structural phase transitions, and prevail in natural and man-made systems^1^. s–s transitions are a central interest in metallurgy and crystallography, but the lack of observations at the single-particle level in the bulk has resulted in many controversies. Their kinetic pathways follow either a diffusive nucleation or a diffusionless martensitic transformation with particles moving in concert^1^,^2^. Martensitic transformations occur widely in alloys^1^, ceramics, minerals, inorganic compounds^3^ and proteins^4^. If the symmetries of the parent and the product lattices have a group–subgroup relation, then the parent lattice deforms continuously into the product lattice without any bond breaking^2^. Such displacive or weak^2^ martensitic transformations often found in shape-memory alloys^5^,^6^,^7^ and giant magnetocaloric materials^8^,^9^; when a group–subgroup relation in symmetries does not exist, the martensitic nucleation involves interparticle bond-breaking. Such reconstructive martensitic nucleation occurs in metals, alloys and some insulating materials^2^. Displacive martensitic transformations often propagate at the speed of sound, while reconstructive martensitic transformations propagate much slower.

Reconstructive s–s transitions are theoretically difficult because an order parameter cannot be defined easily without a group–subgroup relation in the lattice symmetries^2^,^10^. s–s transitions are also challenging to simulate due to the sluggish dynamics^11^. To accelerate the dynamics, simulations were usually performed in small systems^12^, under pressure gradients or even shock waves^13^ to overcome the high free-energy barriers. Experimentally, s–s transitions have been studied by calorimetry^14^, X-ray scattering^15^, acoustic emission^16^ and transmission electron microscopy^17^,^18^, but these techniques cannot resolve the initial stage of nucleation due to the small spatial and timescales. Consequently, the microscopic kinetics of their transition pathways and associated mechanisms remain poorly understood. A central unanswered question about s–s transitions concerns whether their kinetic pathways follow a martensitic or a diffusive nucleation process^11^,^15^,^17^,^19^.

Colloids are outstanding model systems for phase transition studies because micron-sized colloidal particles can be imaged directly by optical microscopy and their thermal motions can be tracked by image processing^20^. Compared with crystallization^21^, melting^22^,^23^ and glass transitions^24^, s–s transitions in colloidal systems have been much understudied^25^,^26^,^27^,^28^,^29^,^30^,^31^. To drive a s–s transition, the colloidal crystal needs to be tunable. For example, an electric-field-induced transition between face-centred cubic (f.c.c.) and body-centred tetragonal (b.c.t.) colloidal crystals was reported to be diffusive for f.c.c.→b.c.t., but martensitic for the reverse b.c.t.→f.c.c. transition^31^. S–s transitions in tunable colloidal crystals have been achieved in small crystallites of DNA-coated colloidal spheres^26^,^32^ and in electric- or magnetic-field-driven s–s transitions^27^,^28^,^30^,^31^. These systems underwent rapid displacive martensitic transformations. The early-stage dynamic processes of the displacive martensitic transformations have not been studied.

Here we study the reconstructive s–s transition between square and triangular lattices, which is one of the simplest s–s transitions without a group–subgroup relation^33^. It occurs within large crystalline domains, and the dynamics process can be well captured. In particular, we examine how a pressure gradient affects the s–s transition, enhances the free-energy barrier crossing and promotes the collective motion of particles, thus resulting in new kinetic behaviours. Phase transitions under pressure gradients represent a simple type of non-equilibrium phase transitions, which are poorly understood yet hugely important for both basic science and technological applications^34^. In fact, most s–s transitions in earth mantel and steel production occur under anisotropic pressure. Here we find rich results about microscopic kinetics in s–s transitions under isotropic or small anisotropic pressures. The major discovery is an early-stage martensitic transformation and a late-stage diffusive nucleation. This result reveals the role of diffusion in the martensitic transformation, which has been discussed in late stage of some martenstic transformations at high temperatures, for example, during the tempering of martensite and in various intermediate structures like Bainite and Widmanstaetten ferrite^1^. We suggest that this pathway could widely exist in atomic systems because once a nucleus formed martensitically, its surface should act like a grain boundary, whereas grain boundaries are known to suppress martensitic transition and promote diffusive nucleation. This hybrid kinetics indicates the breakdown of an empirical criterion of martensitic transformation, that is, the special angle between the parent and product lattices are not necessarily indicating a pure martensitic transformation. Some concepts and terminologies in metallurgy are explained in Supplementary Note 1 since they are seldom used in soft matter physics.

## Results

### Sample preparation and experimental set-up

The tunable colloidal crystals were composed of poly(*N*-isopropylacrylamide) (NIPA) microgel spheres whose diameter, *σ*, linearly changes from 0.76 μm at 26.4 °C to 0.67 μm at 30.6 °C (Supplementary Fig. 2a)^22^. These short-range repulsive spheres (Supplementary Fig. 2b)^29^ exhibit nearly the same phase behaviour as hard spheres^22^,^23^. When spheres are confined between two parallel plates, they can self-assemble into a cascade of crystalline phases as the plate separation *H* increases: 1Δ; 2✓; 2Δ; 3✓; 3Δ; ⋯ (refs 35, 36). Here 1Δ denotes monolayer triangular lattice, 2✓ denotes two-layer square lattice and so on. Similar structures have been observed in plasmas^37^ and electron bilayers in semiconductors^38^. The phase behaviour is determined by the volume fraction *φ* and *H*/*σ* (refs 35, 36). By tuning *σ* with the temperature, both *H*/*σ* and *φ* will change, resulting in an *n*✓→(*n*−1)Δ transition along a tilted path in the phase diagram (Supplementary Fig. 3)^36^.

We heated the interior of a grain with the square lattice using a beam of light with a typical heated area of 10^5^ particles per layer (Supplementary Fig. 4)^23^. We studied the homogeneous s–s transition in a locally defect-free region, or the heterogeneous s–s transition near a dislocation, in a grain boundary or in a triple junction of grain boundaries by choosing a heated area with the desired defects. By contrast, s–s transitions in atomic systems are usually under certain defect densities of various defects, thus single-defect effects and homogenous nucleation are difficult to study. The heated area in the focal plane equilibrated to a temperature *T*_amb_+*δT*, where the ambient temperature *T*_amb_ was tuned with 0.1 °C resolution using the temperature controller and *δT*=1.6 °C is the local optical heating effect, which can be reached in 3 s after switching on the light (Supplementary Fig. 2c)^23^. This sudden change in temperature produced a superheated metastable ✓-lattice. We monitored its evolution towards the equilibrium Δ-lattice under a constant *T* (that is, at constant *φ* and *H*/*σ*). The s–s transition can occur when *T*_amb_<*T*_s−s_<*T*_amb_+*δT*<*T*_m_ (that is, *φ*_amb_> *φ*_s−s_>*φ*_amb_+*δφ*>*φ*_m_), where *T*_m_ and *T*_s−s_ correspond to the melting and s–s transitions, respectively. The heating effect is uniform enough in the central *π*(38 μm)^2^ area of the focal plane (Supplementary Fig. 4) and in the *z* direction for such thin samples^23^.

In most experiments, the colloid was partially filled in the sample cell with air trapped in the corners. Gently pressing one corner induced a tiny drift (flow rate *ν*<0.1 μm s^−1^) of the crystal, which lasted for approximately an hour and slowly varied over several minutes (Supplementary Fig. 5). The crystal drifted as a whole without interlayer gliding under such a low *ν*. We studied the s–s transitions under different *ν* in 50 experimental trials and consistently obtained three types of kinetics in three parameter regimes. In some of the experiments, we applied a better controlled flow using a microfluidic device shown in Supplementary Fig. 10 and obtained similar kinetic pathways. Similar behaviours were observed in *n*✓→(*n*−1)Δ transitions for different *n*. When nuclei are larger than *H*, their shapes were roughly uniform in the *z* direction, and so we mainly monitored the surface layer. We record particle motions using a charge-coupled device camera at 10 frames per s. Particle positions were tracked from the image analysis^39^. Experimental details are provided in the Methods.

### Nucleation inside crystalline domains

Previously we reported the s–s transition in such colloidal thin films under isotropic stress^29^. The transition exhibited a two-step nucleation pathway: *n*✓→liquid nucleus→(*n*−1)Δ, which was confirmed in simulation^40^. This pathway is favoured because a small nucleus is dominated by the interfacial energy instead of the bulk chemical potential, and forming s–s interfaces costs more energy than solid–liquid interfaces. Recently, the intermediate liquid nucleus has been observed in a three-dimensional (3D) atomic crystal^41^, which confirmed that the mechanism is general for both colloidal and atomic systems.

**Figure 1:**
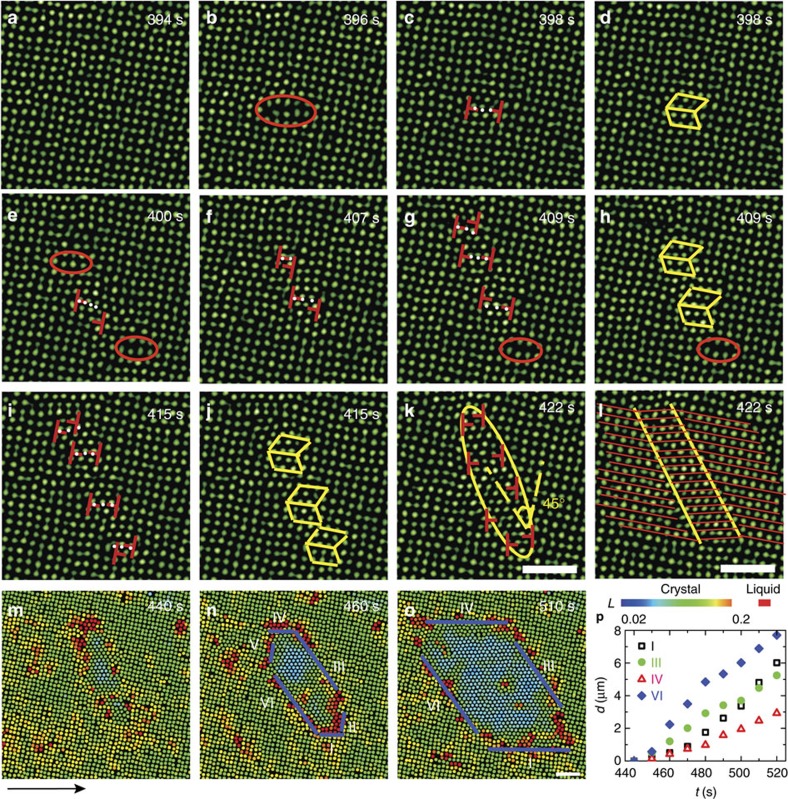
Flow-induced early-stage martensitic precursors and later-stage diffusive nucleus growth. The 5✓→4Δ transition occurred at a locally defect-free region. The generated dislocations are labelled with 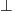. The flows were along the *x* direction of the images (labelled by the arrow below **m**) and moved at *ν*=11 nm s^−1^. Optical heating commenced at *t*=0 s. (**a**–**o**) correspond to Supplementary Movies 1 and 2, respectively. (**a**) A defect-free region in the 5✓-lattice. (**b**) A small gap marked by the ellipse in red developed under thermal fluctuation at *t*=396 s. (**c**) A row of three particles labelled in white moved from the bulk layer to the gap in the surface layer, forming a pair of dislocations. (**d**) The same image as **c** can be viewed as a very small Δ-lattice precursor labelled by the yellow ‘butterfly'. The dislocations glided in opposite directions and oscillated at ∼0.2 Hz with an amplitude of two lattice constants, then generated new gaps and new dislocation pairs alternatively around the first pair. All the dislocation pairs were arranged in a line and oscillated in phase during the nucleation precursor stage **e**–**i**. From **i**–**l**, the dislocation pairs stopped oscillating and martensitically formed an elliptical Δ-lattice nucleus. In **l**, the 2 yellow lines indicated the habit planes and the 13 red lines are along the lattice orientations in the parent and product phases. The martensitic nucleus then grew in all directions through diffusion in **m**–**o**. The colour bar above **p** shows different values of the dynamical Lindemann parameter *L* used in **m**–**o**. Liquid-like particles are defined as particles with *L*>0.2 and low bond-orientational orders and are labelled in red^23^ (Methods). They swapped positions with neighbouring particles, hence they are liquid, not glass. Scale bars, 5 μm. The nucleus developed facets in **n** and grew into a parallelogram shape in **o**. The Δ-lattice had one layer less than the ✓-lattice in the *z* direction, hence it was more compact in the *xy* plane with lower Lindemann parameters and about 3% lower lattice constant as the nucleus grew larger. (**m**–**o**) Coloured by Lindemann parameter *L*. Red colour represents liquid-like particles, which *L*>0.2 and low bond-orientational order parameter (Methods)^29^. (**p**) Displacements of the four facets along their normal directions after subtracting the background flow.

Here we observed that the intermediate liquid nucleus vanished under a small flow of merely *ν*≳10 nm s^−1^ (Fig. 1). After a long incubation period in a defect-free region, a row of several particles shifted from the bulk layer to the surface layer forming a pair of dislocations with opposite Burgers vectors. The newly inserted row was always oriented along the[10] or [01] direction of the ✓-lattice and tended to be aligned with the direction of the flow. The two dislocations glided and oscillated in opposite directions perpendicular to the inserted row of particles, which produced a few more dislocation pairs. These dislocation pairs formed in parallel and arranged in a line (Fig. 1). Interparticle bonds broke when new rows of particles were inserted (that is, plastic deformation) but not when the dislocation pairs oscillated (that is, elastic deformation). Each inserted row of particles distorted the local ✓-lattice to a Δ-lattice, forming a tiny twinning structure labelled by two mirror-symmetrical parallelograms in Fig. 1d. The twinning structure did not grow large because it will induce strain energy in the parent phase. These disconnected Δ-lattice ‘butterflies' vanished and born as the dislocation pairs oscillated, thus we called the dislocation pairs as nucleation precursor rather than nucleus. Generally, before the nucleus forms, the parent phase would often develop a certain structure that would reduce the free-energy barrier and trigger the nucleation. Such a nucleation precursor has attracted substantial interest in the studies of crystallization^42^,^43^, melting^23^ and s–s transitions^44^. For example, elastic lattice softening has been detected in Ti–Ni-based alloys before martensitic transformations^44^, but the structure and dynamics of this precursor have not been observed at the microscopic scale. In the two-step nucleation under a negligible flow (*ν*<10 nm s^−1^), we previously found that the precursors were in fact particle-swapping loops rather than the conventionally assumed crystal defects^29^. Here we found that the precursors at *ν*>10 nm s^−1^ were oscillating dislocation pairs, which represent a new type of nucleation precursor. Such martensitic type of precursor has not been suggested before. Here we call it martensitic precursor, which is beyond the conventional structural description (for example, vacancies^45^ or dislocations^46^ in melting; dense^42^ or ordered^43^ blobs in crystallization) of nucleation precursors.

**Figure 2:**
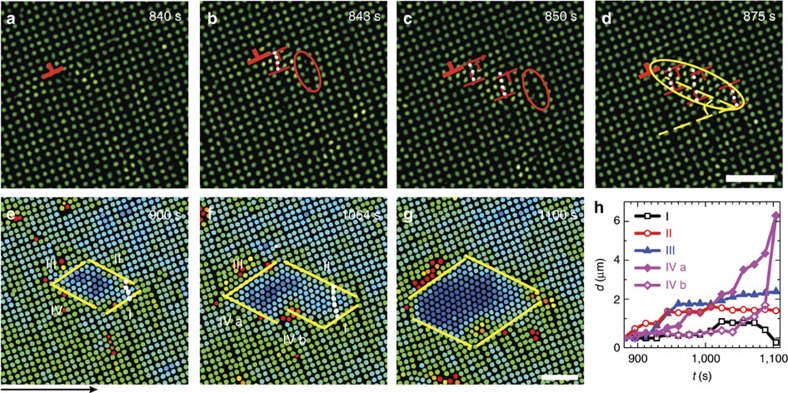
The nucleation process near a pre-existing dislocation. (**a**–**g**) correspond to Supplementary Movies 3 and 4, respectively. A row of four bulk particles were inserted into the surface layer and formed a dislocation pair on the low-density side of the dislocation after 843 s of incubation. The dislocation pair oscillated and induced more pairs, resulting in an elliptical Δ-lattice nucleus, which grew into a parallelogram shape through both diffusion and the collective insertion of rows of particles (coloured in white). After 1,100 s, the nucleus growth was purely diffusive. The Δ-lattice was oriented along the major axis of the ellipse and at 45° relative to the ✓-lattice in **d**. Colours in **e**–**g** represent the Lindemann parameters as in Fig. 1m–o. (**h**) Displacements of the four facets along their normal directions after subtracting the background flow. When the nucleus became large, the diffusive growth (solid symbols) dominates over the collective insertion of rows of particles (open symbols). From **f**–**g**, facet IVb became shorter and eventually vanished as facet I propagated to the left corresponding to its negative slope near 1,100 s in **h**. Scale bars, 5 μm.

**Figure 3:**
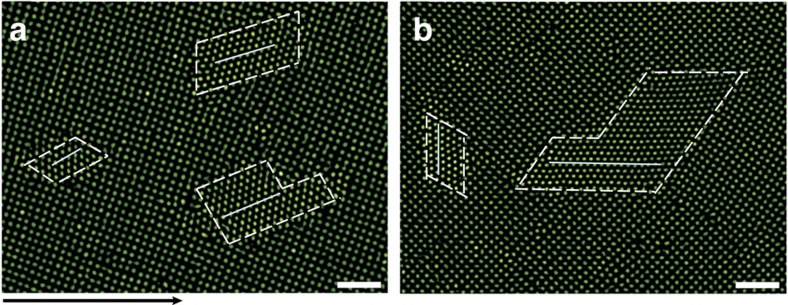
Multiple nuclei formed within one crystalline domain. The early-stage martensitic nucleation dictated the lattice orientation of all the Δ-lattices to be 45° relative to the ✓-lattice. The lattice orientations of the Δ-lattices are parallel (that is, 45°−45°=0°) in **a** and perpendicular (45°+45°=90°) in **b**. The small angles between the Δ-lattices in **a** are due to the defects and the nucleus-expansion-induced lattice distortion in the ambient ✓-lattice. The flows were along the *x* direction of the images (labelled by the arrow below **a**). Scale bars, 5 μm.

**Figure 4:**
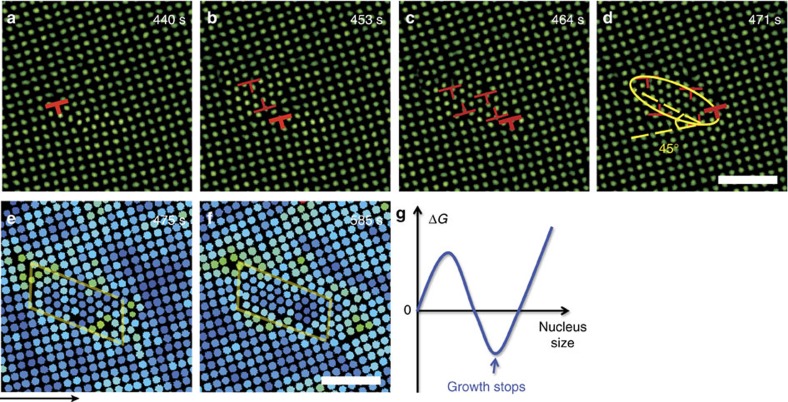
Incomplete martensitic nucleation in 5✓→4Δ transition at high volume fraction *φ*. The flow *ν*=22 nm s^−1^ is along the *x* direction (Supplementary Movie 6). The blue colours in **e**,**g** reflects low Lindemann parameter values, that is, high *φ*. Scale bars, 5 μm. The climbing and gliding of the pre-existing dislocation labelled with a red 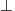 in **a** induced a dislocation pair to form in **b**. The original dislocation kept climbing and gliding, inducing another pair of dislocations in **c** and producing an elliptical Δ-lattice nucleus in **d**. The nucleus grew into a parallelogram shape and stopped growing thereafter. Colours in **e**,**f** represent the Lindemann parameters as in Fig. 1m–o. (**g**) Schematic of the free energy Δ*G* of the nucleus in such incomplete martensitic nucleation. The nucleus stopped growing at the minimum Δ*G*. Δ*G* increases with the nucleus size due to strain induced by the nucleus expansion.

From Fig. 1i–l, dislocation pairs collectively and rapidly distorted the ✓-lattice into a Δ-lattice (Fig. 1k,l) as an in-plane displacive martensitic nucleation without any bond breaking. The resulting nucleus was elliptical in the *xy* plane with a uniform shape along the *z* direction, and the habit planes were labelled by the yellow lines in Fig. 1l. Similar dislocation-pair distributions on the elliptical nucleus surface have been observed in atomic systems^1^,^47^, but their kinetics and formation processes are not available. Here we observed that multiple dislocation pairs in the precursor stage acted like a post-critical nucleus: they irreversibly induced more dislocation pairs to form leading to an elliptical post-critical nucleus. In contrast, the critical size of a liquid nucleus is about 50–100 particles per layer in the two-step diffusive nucleation under no flow^29^. Besides the collective formation and oscillation of dislocation pairs, this nucleation precursor process exhibited another four features of martensitic transition^1^: (1) particles were aligned in parallel lines across the nucleus surface as shown in Fig. 1l; (2) the displacement of each particle was within one lattice constant; (3) the elliptical nucleus shape has a much higher aspect ratio than those of diffusive nucleation^1^,^29^; and (4) the lattice orientations of all the nuclei followed a particular angle relative to the parent lattice (that is, 45° or [10]_✓_ || [11]_Δ_ in Figs 1, 2, 3, 4). In addition, the major axis of the elliptical nucleus was always at 45° relative to the ambient ✓-lattice (Figs 1k and 2d).

The later stage of post-critical nucleus growth is crucial to the phase transition speed and final microstructures. The martensitic nucleation in Fig. 1a–l yielded a post-critical nucleus, which rapidly grew via the attachment of individual diffusive particles without collective motions (Fig. 1m–o). The crossover from the early-stage martensitic nucleation to the later diffusive growth is not sharp at high *φ*; rows of particles were collectively inserted onto the nucleus' tips and other particles individually attached to the nucleus' surface (Fig. 2e,f and Supplementary Movie 3). When the nucleus grew large, the growth became purely diffusive (Fig. 2g) because the energy barrier is higher to collective inserting a long row of particles into a larger nucleus.

As the elliptical nuclei grew diffusively, their aspect ratios decreased and they developed facets (Figs 1m–o and 2e–g). All the nuclei surfaces propagated at speeds below 100 nm s^−1^ (for example, Figs 1p and 2h), which is much lower than the speed of sound ∼0.1 m s^−1^ in typical colloidal crystals^48^. This is a feature of diffusive nucleation and distinct from the martensitic growth, which usually grow at a much higher speed close to that of sound^1^. We observed that the facet propagated more slowly at a higher *φ*, for more coherent facets, or downstream of the flow (see the slopes in Figs 1p and 2h). Typically a nucleus first developed six facets that were usually at 45° and 15°(=60°−45°) relative to the ✓-lattice, hence they were incoherent, that is, the two lattices did not completely match at the interface (Supplementary Fig. 1). By contrast, coherent interfaces with 0° mismatch typically formed in nuclei at grain boundaries and triple junctions. The mismatch of incoherent interfaces provides space for particle rearrangement, hence incoherent interfaces propagate much faster than coherent interfaces (Supplementary Movie 5)^1^,^29^. This can also be explained in terms of energy: coherent interfaces have the lowest energy, and thus it is more difficult to from high-energy kinks to trigger interface propagation. In addition, we found that incoherent facets propagated at similar speeds under an isotropic stress without flow^29^, but exhibited different speeds under a flow. In Figs 1m–o and 2e–g the upstream particles have higher Lindemann parameter *L* (that is, lower *φ*), and thus have more space to rearrange themselves and transform into a Δ-lattice. Consequently, facet VI propagated faster than facet III in Fig. 1n,p, and facet IV propagated faster than facet II in Fig. 2f,h. Facets I and IV in Fig. 1n were along the flow direction, thus they extended quickly along the lateral direction and propagated slowly along the normal direction. Note that a fast lateral growth corresponds to a slow normal growth of this facet and fast normal growth of neighbouring facets and vice versa^1^,^29^. This is similar to the observed higher mobilities of grain boundaries perpendicular to an external pressure^49^. *L* near facet I was higher than that near facet IV, thus facet I grew faster (Fig. 1n–p). Facets II and V in Fig. 1n were almost perpendicular to the flow, thus they grew rapidly along their normal directions and slowly along their lateral directions. Consequently, they were eventually overwhelmed by neighbouring facets and the nucleus took the shape of a parallelogram. In addition, the early history, for example, whether the nucleation was from a pre-existing defect (Fig. 2a) or via a two-step nucleation with an intermediate liquid state (Supplementary Fig. 7), did not affect the facet propagation speed.

A large crystalline domain usually contained multiple nuclei (Fig. 3). For two-step diffusive nucleation with an intermediate liquid nucleus at *ν*<10 nm s^−1^, the lattice orientations of nuclei are mainly random, a feature of diffusive nucleation^17^. For nucleation at *ν*>10 nm s^−1^, the nucleus lattices exhibit a 45° angle relative to the parent lattice (that is, [10]_Δ_ || [11]_✓_) due to the early-stage martensitic precursor (Fig. 3).

The definitions of martensitic and diffusive transformations are based on the dynamic motions of particles, which are difficult to observe in bulk atomic crystals. Therefore, martensitic transformations are often identified indirectly from static structures such as the angle between the parent and product lattices. For example, by quenching the crystal during the transformation and cutting up the bulk, the lattice orientations of the frozen nuclei at the exposed cross-section can be observed by electron microscopy^17^. If the orientations between the parent and product lattices exhibit a special relation (for example, [10]_Δ_||[11]_✓_ in Figs 1, 2, 3, 4), then it is identified as a martensitic transformation; if the mismatch angles are random, then it is a diffusive nucleation^1^,^17^. We found that this empirical criterion based on crystallographic relationship for martensitic transition may not always hold because a rapid martensitic precursor can induce a special mismatch angle, even though the nucleation process is mainly diffusive (Figs 1, 2, 3). Note that the lattice mismatch angle cannot be changed easily after the embryo has formed because a high energy barrier must be overcome to rotate the whole nucleus. Moreover, we conjecture that a reconstructive martensitic nucleation could later change into diffusive growth in atomic crystals for three reasons. First, once a nucleus forms, the transformation at its incoherent surface should be diffusive since a collective lattice distortion is difficult in geometry. Second, in metallurgy martensitic transformations generally do not occur at grain boundaries^1^,^17^. Surfaces of nuclei have similar structures to grain boundaries and thus should have similar effects. Third, in some atomic martensitic transformations, the aspect ratios of newly formed nuclei are usually very high but are reduced as the nuclei grow^1^. This could be attributed to the diffusive growth, which is rather isotropic compared with the anisotropic martensitic deformation. Before nuclei form, there is no s–s interface inside crystalline domains, hence the nucleation tends to occur martensitically since it is not easy for particles to diffuse individually in a defect-free region. After the nucleus forms, its incoherent interfaces promote the diffusive nucleation. This mechanism of early-stage martensitic and later-stage diffusive nucleation is expected to be independent of colloidal and atomic systems.

**Figure 5:**
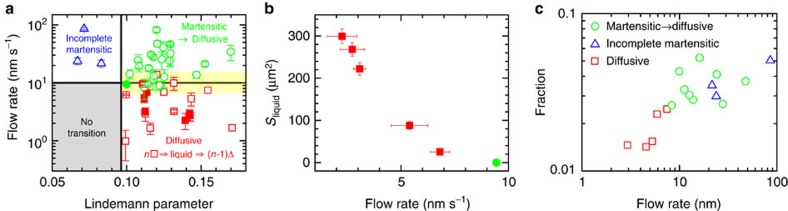
Nucleation pathways within a crystalline domain at varied flow rates. (**a**) Two-step diffusive nucleation (squares, Supplementary Fig. 7 and ref. 29), martensitic precursor followed by diffusive growth (circles, Figs 1, 2, 3 and Supplementary Movies 1–4) and incomplete martensitic nucleation (triangles, Fig. 4 and Supplementary Movie 6) in different parameter regimes. The error bars of flow rates in **a**,**b** indicate the fluctuations during the incubation periods. The yellow band marks a crossover region due to thermal fluctuations and different local dislocations. The five red solid squares and the green solid circle in **a** are also shown in **b**. No s–s transition occurs at *L*<0.1 (that is, high *φ*) and *ν*≲10 nm s^−1^. (**b**) The maximum area of the liquid nucleus in the two-step diffusive nucleation^29^ decreases with the flow rate. The six data points were measured in the same sample region with the same *H* in different experimental trials. The same sample region was reused by cycling the temperature to induce the Δ-lattice nucleus to grow and shrink. Different samples with slightly different *H* and *φ* yielded similar threshold *ν*. The error bars of liquid areas represent the uncertainties in the measurements of the largest liquid nuclei. (**c**) A higher flow rate results a higher fraction of large-strain (|*Γ*|>0.2) particles.

At high *φ*, particles have little space to diffuse or move concertedly to form a nucleus. Consequently, s–s transitions can hardly occur unless the driving force is high (that is, high *ν*) as shown in Figs 4 and 5a. At high *φ* and high *ν*, the early-stage nucleation was similar to that in Fig. 1, but the nucleus stopped growing at about 50 particles per layer (Fig. 4). Such incomplete martensitic transitions also exist in atomic systems, which are usually caused by large stresses at low temperatures away from grain boundaries^1^. Our colloidal experiments confirmed that incomplete nucleation was only observed within crystalline domains at high *φ* (that is, low *T* for atomic system) and high *ν* (Fig. 5a). Martensites in such incomplete transitions, also called hardenites, make the grain size finer and thus enhance the strength of the solid^1^; hence incomplete martensitic transitions are widely used in steel hardening. The nucleation is incomplete because the expansion of a larger nucleus induces more strain in the parent lattice, that is, a higher free energy Δ*G* for a larger nucleus. Consequently, the nucleus stopped growing at the minimum Δ*G* as illustrated in Fig. 4g. Similar Δ*G* curves have recently been found in the 3D crystallization of DNA-coated colloidal spheres^50^ and in the two-dimensional (2D) crystallization on a curved surface^51^.

Figure 5b shows that the maximum area of the intermediate liquid nucleus in the two-step nucleation can be hundreds of particles per layer. These particles are clearly liquid-like since they actively swapped positions with neighbouring particles. The liquid nucleus size decreased with *ν* and vanished at *ν*≳10 nm s^−1^ (Fig. 5b). Although such a small *ν* could hardly induce any discernable layering in liquid^52^, it effectively promoted the formation of a Δ-lattice and suppressed the liquefaction of the ✓-lattice, which presents new challenges to theory. Although our observed 10 nm s^−1^ flow and the estimated pressure gradient 0.3 Pa m^−2^ are very small, they could have significant effect for colloidal crystals because they are much softer than atomic crystals. If the elastic modulus of an atomic crystal is 10^10^ times larger than our colloidal crystal, we expect that a 10^9^ Pa m^−2^ pressure gradient can help to align liquid particles with a better order and suppress the intermediate-stage liquid in atomic crystal.

**Figure 6:**
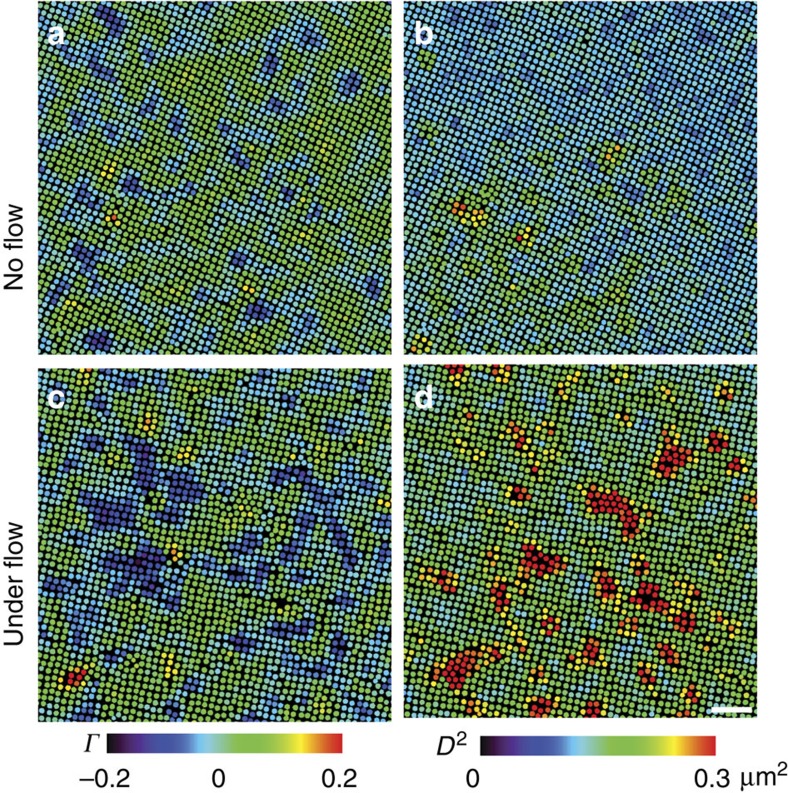
Affine and non-affine strain fields. The superheated five-layer ✓-lattice crystals without a flow (**a**,**b**; that is, the sample in Supplementary Fig. 7) and under a flow (**c**,**d**; that is, the sample in Fig. 1 and Supplementary Movie 7). (**a**,**c**) are coloured by the shear strain *γ* and **b**,**d** are coloured by the non-affine parameter *D*^2^. Scale bar, 5 μm.

**Figure 7:**
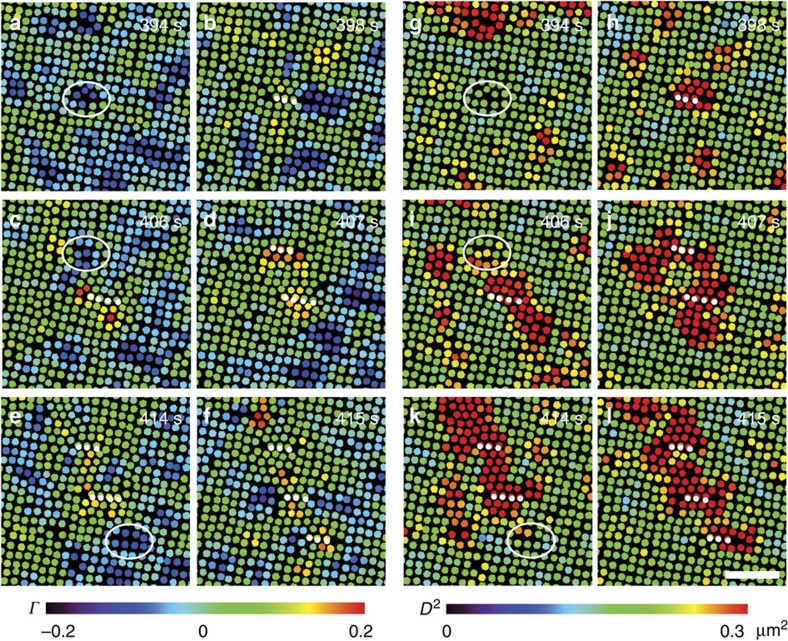
The evolution of strain field in the formation of dislocation pairs nucleation shown in> Figure 1. (**a**–**f**) are coloured by the shear strain *γ*. (**g**–**l**) are coloured by the non-affine parameter *D*^2^. Newly inserted particles are labelled by white dots and their precursors are highlighted by the white ellipses. Scale bar, 5 μm.

The mechanical effect of a flow is revealed by the flow-induced strain field (Fig. 6). The local strain is determined from the structural distortion of the nearest neighbours of the particles^53^. The optimized affine deformation tensor of particle *i*, 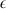
_*i*_, is determined by minimizing the mean square difference 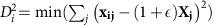 (ref. 53). **x**_**ij**_ is the vector from particle *i* to its nearest neighbours *j*. **X**_**j**_ is the vector to particle *j* in its ideal square lattice site. The symmetric part of 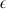 corresponds to the local strain tensor of the particle. Here we use the shear component of the strain *Γ*=2
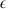
_*xy*_ on the *xy* plane to quantify the affine deformation and the non-affine parameter *D*^2^ to reflect the local deviation from affine deformation^19^,^53^,^54^. The fraction of large-strain particles increases with the flow rate (Fig. 5c), indicating that the flow-induced large shear strain assisted the generation of dislocation pairs and suppressed metastable liquid nuclei. The strain fields about *Γ* and *D*^2^ are rather uniform and small in space-time under no flow, and fluctuated strongly with large-strain clusters under a flow (Fig. 6). Large shear strain clusters highlighted by ellipses in Fig. 7a,c,e triggered the formation of dislocation pairs, and induced plastic deformations associated with large non-affine strain (red particles in Fig. 7g–l. If the dislocation pairs did not form, that is, without a row of inserted particles, then the region of large affine strain relaxed to ✓-lattice rapidly (Supplementary Movie 7).

The dynamics of non-affineness can determine the nucleation kinetics^55^. If the propagation speed of non-affineness is fast, that is, of the same order as the nucleation kinetics, then the bond-breaking in the non-affineness transformation induces a diffusive nucleation. On the other hand, the transformation is martensitic if the nucleation front driven by the affine strain is faster than the propagation of non-affineness. In the initial stage of our s–s transitions under flows, the affine strain occurred before the non-affine part and propagated anisotropically (Fig. 7), thus the transformation was martensitic; whereas at the later stage, the non-affine strain propagated at the same speed of the nucleus growth (Supplementary Fig. 9), thus the transformation was diffusive.

### Nucleation near dislocations

Pre-existing defects strongly affect phase transition kinetics. Dislocations are expected to promote martensitic transformations due to their elastic strain energy^1^. The densities of martensitic nuclei and pre-existing dislocations were found to be proportional in metals and alloys^1^, but the effect of a single dislocation is unclear. Here we observed that nucleation had a higher chance near a single pre-existing dislocation than in a defect-free region because of a lower free-energy barrier. The nucleation kinetics was similar to that in the defect-free region, that is, two to five pairs of dislocations were formed via concerted motions followed by diffusive growth (Fig. 2 and Supplementary Movies 3 and 4). An edge dislocation can be viewed as a perfect crystal missing a half plane of particles. Thus, the density is lower on one side of the dislocation. We observed that the inserted rows of several particles were always on the lower density side and parallel to the missing half plane of particles (Fig. 2) in different samples regardless of the flow direction. The motion of a pre-existing dislocation triggered the insertion of a row of particles at a few lattice constants away.

### Nucleation at grain boundaries

**Figure 8:**
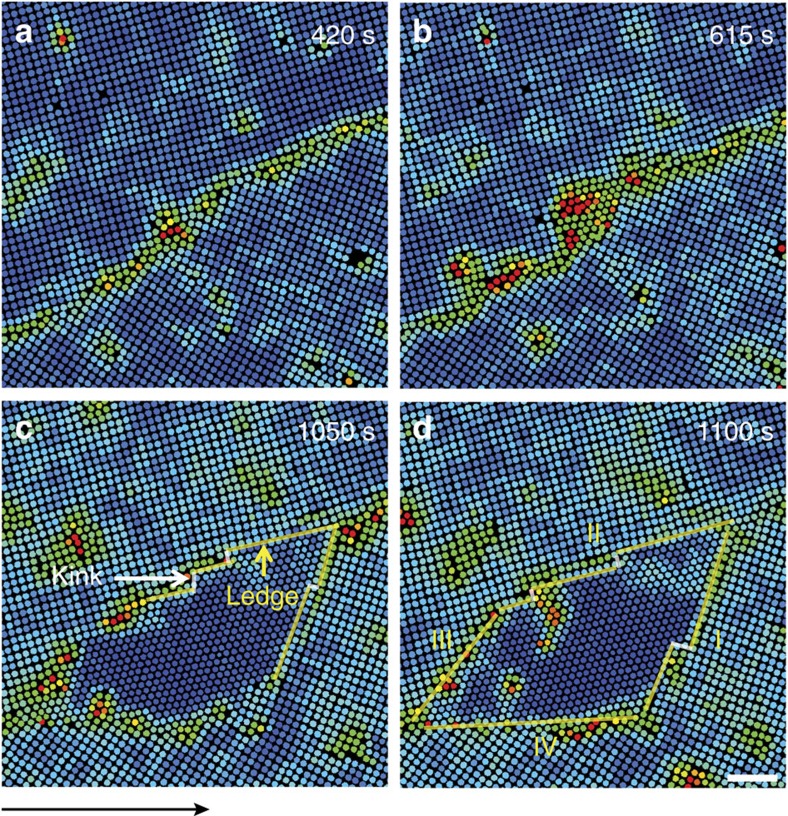
5✓→4Δ diffusive nucleation at a grain boundary. The flow was along the *x* direction of the images (labelled by the arrow below **c**) with 〈*ν*〉=36 nm s^−1^ (Supplementary Movie 8). Colours represent Lindemann parameters as in Fig. 1m–o. Optical heating commenced at *t*=0 s. Scale bar, 5 μm. (**a**) The incubation stage. (**b**) At *t*=415 s, red particles on the grain boundary swapped positions with their neighbours and formed a Δ-lattice nucleus. (**c**) The nucleus grew through particle diffusion and developed facets. (**d**) At 1,100 s, the nucleus grew into a polygon with coherent facets I and II and incoherent facets III and IV at 45° relative to the ✓-lattice. The kinks on the coherent facets originated from particle diffusions at the grain boundary.

Most crystals contain numerous grain boundaries and triple junctions, hence their effects on the s–s transition are particularly important. We found that the nucleation at grain boundaries and triple junctions can exhibit different kinetics. When *ν*≲10 nm s^−1^, the s–s transition at a grain boundary or an asymmetric triple junction is similar to that inside a domain, that is, it follows a two-step diffusive nucleation with an intermediate liquid nucleus^29^. When *ν*≳10 nm s^−1^, however, the transition at grain boundaries becomes a one-step diffusive nucleation without the intermediate liquid nucleus (Fig. 8). The early-stage martensitic nucleation was absent from grain boundaries and triple junctions, which is consistent with the behaviours in atomic systems^1^,^17^. Grain boundaries acted as a sink for small defects, hence their nearby lattices were highly ordered without distortions and strains. Consequently, they did not promote martensitic transformations. On the other hand, *L* within four layers of a grain boundary were higher^22^, thus promoting the diffusive nucleation. The nucleation occurred at the grain boundary rather than at the nearby dislocations or in the defect-free regions in Fig. 8, demonstrating that the nucleation barrier at the grain boundary was lower. Figure 8 shows a lower *L* (that is, higher *φ*) but a shorter incubation time than does Fig. 2, indicating that the nucleation occurred more easily at the grain boundary than inside the domain.

As the nucleus grew larger, it developed two coherent facets for a lower interfacial energy. The other two incoherent facets oriented 45° away from the square lattice. Such a 45° inclination angle (Supplementary Fig. 1) can be seen in Figs 1, 2, 3, 4 and 8, indicating that such facets have a relatively lower interfacial energy. Grain boundaries with different mismatch angles and inclination angles (Supplementary Fig. 1) caused nuclei to take different polygonal shapes, with most having incoherent facets and one or two coherent facets. Incoherent facets (for example, facets III and IV in Fig. 8c) are rough. Thus, particles from the parent phase can easily transform into the product phase and induce nucleus growth. In contrast, the coherent facets tended to maintain a low-energy flat shape. Thus, they had few sites to attach particles and propagated slowly. The coherent facets (for example, facets I and II in Fig. 8c) started propagating mainly from the junctions of grain boundaries where particles diffused onto the coherent facet and formed ledges and kinks (Fig. 8c). ✓-lattice particles tended to transform into Δ-lattice particles at the kinks, resulting in the growth of ledges and propagation of coherent facets. Such microscopic growth kinetics has not been proposed or observed before, but could exist in atomic systems because the mechanism is simple and should not be limited to colloids.

### Nucleation at a symmetric triple junction

**Figure 9:**
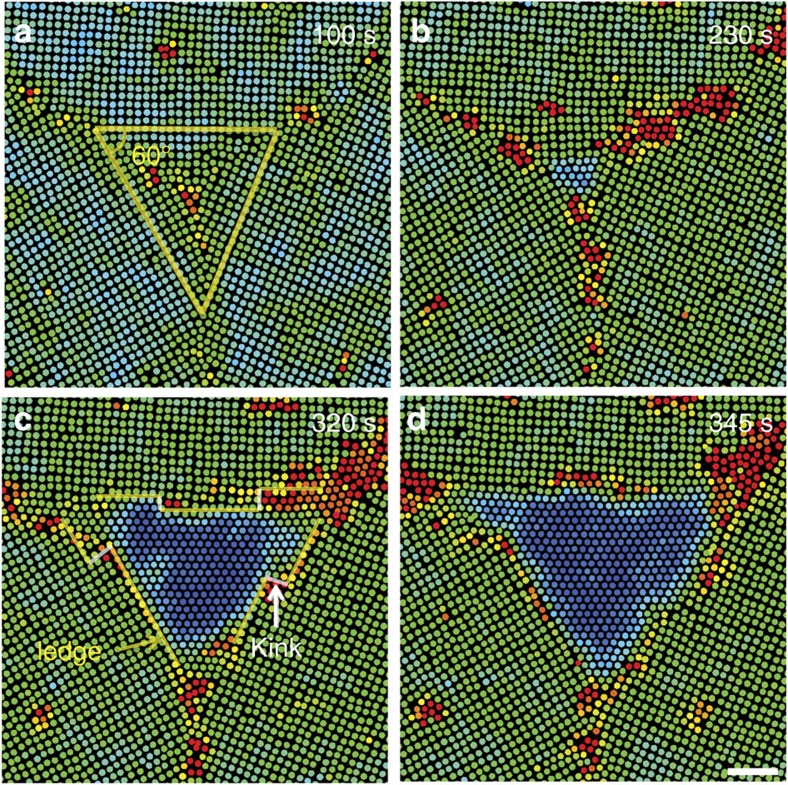
5✓→4Δ diffusive nucleation at a symmetric triple junction. *ν*=2 nm s^−1^. Colours represent the Lindemann parameters as in Fig. 1m–o (Supplementary Movie 9). Optical heating commenced at *t*=0 s. Scale bar, 5 μm. (**a**) The mismatch angles between the three lattices are all ∼60°, so that all facets are coherent for a triangular-shaped Δ-lattice nucleus. (**b**–**d**) The nucleus growth was mainly initiated by particle rearrangement at the three corners, followed by the development of kinks (labelled in **c**), which served as the growth front.

We found that applying a small flow is not the only way to suppress the intermediate liquid nucleus during the ✓−Δ transformation. Figure 9 shows a Δ-lattice directly nucleating at a triple junction through the diffusion of individual particles without forming an obvious liquid nucleus even at *ν*≲10 nm s^−1^. The mismatch angles between the neighbouring lattices were all 60°. An equilateral triangle shape could make all nucleus' facets coherent for a lower energy. Since the interfacial energies *γ*_coherent_<*γ*_liquid_<*γ*_incoherent_ in this colloidal system^29^ and most metals and alloys^1^, a Δ-nucleus whose facets are all coherent is more energy favourable than a liquid nucleus and can form via overcoming a lower energy barrier. By contrast, a Δ-lattice nucleus embedded in a single ✓-lattice domain (Supplementary Fig. 1b) or at an asymmetric triple junction, the nucleus facets cannot all be coherent. Consequently, the nucleation is a two-step process with a small intermediate liquid nucleus at low flow rates (Supplementary Figs 3 and 7 of ref. 29). In Fig. 9, the strong particle rearrangements featuring a high *L* mainly occurred at the junction of the nucleus and grain boundaries rather than on the nucleus facets because the facets were coherent and tended to be flat. Particles were attached to the coherent facets from the junctions of grain boundaries and formed high-energy kinks, which induced nucleus growth, similar to those in Fig. 8.

## Discussion

All homogeneous and heterogeneous *n*✓→(*n*−1)Δ transitions follow the nucleation process, which is a signature of the first-order transition. The rich behaviours of different nucleation pathways are summarized in Table 1. These phenomena were well reproduced in many trials of experiments such as those shown in Fig. 5a. These results show that a small anisotropic stress can effectively suppress the intermediate liquid state, promote martensitic transition at the early stage and affect the growth speed of the nucleus facet, nucleus shape and lattice orientation. Different defects induce different nucleation rates and facet coherencies. Therefore, applying stress and controlling defect density provide effective ways to manipulate the s–s transition and microstructure evolution in practical applications. For example, Figs 8 and 9 contain more coherent interfaces than Figs 1, 2, 3, 4 and Supplementary Fig. 7, thus using a polycrystal with smaller grain sizes and more grain boundaries can induce more coherent interfaces, which have low interfacial energy^1^. Applying a pressure gradient can effectively align the lattice orientations of the nuclei, which can tune the strain in the parent lattice to form nuclei of desired shapes because a disk-shaped nucleus minimizes the strain energy whereas a spherical nucleus minimizes the interfacial energy.

The following five phenomena observed in atomic crystals have been confirmed with single-particle dynamics in our colloidal crystals: first, anisotropic stress promotes s–s transition (Fig. 5a)^17^. Second, the nucleation is diffusive under an isotropic stress whereas it became martensitic under an anisotropic stress^15^. Third, nuclei stop growing at low temperatures (high *φ* for colloids), that is, incomplete martensitic nucleation (Fig. 4)^1^; fourth, incoherent interfaces propagate faster than coherent interfaces (Supplementary Movie 5); and fifth, defects associated with a strain field such as dislocations promote early-stage martensitic transformations (Fig. 2), while defects without strains such as grain boundaries promote diffusive nucleation (Figs 8 and 9)^1^. These phenomena exist in both 3D atomic crystals and thin-film colloidal crystals, indicating that the colloid is a good model system for atomic s–s transitions.

Moreover, the direct *in situ* observation enabled us to resolve five new phenomena that can hardly be observed in atomic crystals. First, particle motions in reconstructive martensitic nucleation have rarely been observed. Here we observed a martensitic embryo formation for the first time (Fig. 1). The generation and oscillation of dislocation pairs teared up the parent crystal and triggered the nucleation. Such a dynamical martensitic precursor is beyond the conventional structural description of precursors in melting, crystallization and s–s transitions. Second, our major result is the following: a special orientational relationship between the parent and product lattices does not necessarily indicate a martensitic transformation because a martensitic precursor itself is enough to yield a special angle even though the nucleation process is mainly diffusive. The later-stage diffusive growth determines the facet growth and nucleus shape, but these factors can hardly be used to distinguish between diffusive and martensitic kinetics. Third, an X-ray diffraction experiment on olivine–spinel transition suggested that the anion sublattice transforms in a martensitic process first and then cations transform via diffusion^15^. It is a new type of transition named as pseudomartensitic transformation, in contrast to the previously assumed purely martensitic and purely diffusive nucleation. Here the observed martensitic precursor followed by diffusive growth demonstrates that even a single-component system of monodispersed hard spheres can exhibit complicated s–s transition kinetics. Fourth, facets downstream of the pressure grow rapidly in the normal direction and slowly in the lateral direction (Fig. 1). Fifth, coherent facets mainly grow from kinks developed from the junctions of the grain boundaries or other facets (Figs 8 and 9). These five phenomena do not depend on particular properties of colloids, thus may also exist in atomic systems. To our knowledge, these five kinetic phenomena have never been observed or suggested in experiment or simulation, and thus can help to refine the theory.

Most phenomena observed in such thin-film colloidal crystals could similarly exist in 3D atomic crystals because the wall effect in thin-film crystals is proportional to the nucleus volume and can be absorbed into the effective bulk chemical potential^29^. For example, the two-step nucleation with an intermediate liquid shown in Supplementary Fig. 7 should similarly exist in 3D crystals because the wall effect can be absorbed into the bulk free-energy term, while the interfacial energy dictates the initial state of the nucleus. In addition, *γ*_coherent_≲*γ*_crystal–liquid_<*γ*_incoherent_ in colloidal crystals^29^ and in most metals and alloys^1^. Consequently, their nucleus formation and growth behaviours should be similar. Although the wall confinement restricts the nucleus rotation in quasi-2D, a nucleus in quasi-2D needs less matching angles to form coherent interfaces than those in 3D. Therefore, the intermediate liquid does not form more easily in quasi-2D than in 3D. In fact, many 3D atomic crystals have no lattice-constant matching and cannot form coherent interfaces at all. By contrast, our thin-film triangular and square lattices have almost the same lattice constant, thus coherent interfaces have been observed. Since the intermediate liquid can form in quasi-2D systems allowing coherent interfaces, it is should be more easily to form in those 3D atomic crystals, which do not have coherent interfaces. Even for 3D atomic crystals, which can have coherent facets, most facets have to be incoherent (see a 2D example in Supplementary Fig. 1b). Consequently, the total interfacial energy of an atomic crystalline nucleus could still be higher than a liquid nucleus because the interfacial energy ranges from 500 to 1,000 mJ m^−2^ for incoherent interfaces, <200 mJ m^−2^ for coherent interfaces and ranges from 30 to 250 mJ m^−2^ for solid–liquid interfaces in most metals and alloys^1^. In fact, the early-stage liquid has been suggested in experiments about 3D metals^41^ and graphite–diamond transition^56^, and observed in the simulation of the s–s transition of hard-sphere thin-film crystals^40^ and 2D ices^57^.

## Methods

### Sample preparation

NIPA (pNIPAM, pNIPA or NIPA) microgel spheres were synthesized and dispersed in an aqueous buffer solution with 1 mM acetic acid. These microgel spheres were slightly charged with short-range steric repulsions (Supplementary Fig. 2). The effective diameter *σ* changed linearly with the temperature *T* as revealed by directly imaging isolated particles stuck to the glass wall in a dilute suspension (Supplementary Fig. 2)^53^. Since the diameter of a soft sphere is ambiguous, we slightly rescaled the measured *σ*(*T*) so that the melting volume fraction of the 3D crystal was the same as that of hard spheres (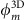
=54.5%). Then the freezing point 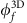
=49%, which was very close to that of hard spheres 49.4% (ref. 23). Therefore, the phase behaviour of the NIPA colloid was very similar to that of the hard spheres. The equilibrium phase diagram of hard spheres confined between two hard walls is shown in Supplementary Fig. 3 from a previous simulation^36^. In previous thin-film crystal melting experiments^58^,^59^ and s–s transition experiments^29^, we found that NIPA spheres have almost the same phase behaviours as hard spheres, 1Δ, 2✓, 2Δ, 3✓, 3Δ, ⋯ as the wall separation increases. By changing *σ*, the transition path of spheres under a given *H* can be calculated as shown in Supplementary Fig. 3. According to the slope of the path, increasing the temperature (decreasing *σ*) resulted in either *n*✓→(*n*−1)Δ→liquid, *n*✓→liquid or *n*Δ→liquid transitions, but not *n*Δ→*n*✓→liquid or *n*✓→*n*Δ transitions. These behaviours were confirmed in our experiments. Here we only focused on the temperature regime near the *n*✓→(*n*−1)Δ transition, which occurs below the (*n*−1)Δ→liquid transition point. For 5✓→4Δ transitions, *H*/*σ*≃3.9 and 4.1, and *φ*≃0.62 and 0.55, before and after the local heating, respectively. The samples were prepared as in our earlier thin-film melting experiments^58^,^59^. A droplet of colloidal suspension was placed between a glass slide and a coverslip. The sample cell thickness *H* was roughly determined by the added volume of colloidal suspension and its spreading area. When the colloidal suspension had spread over approximately half of the 18 × 18 mm^2^ coverslip area, we hermetically sealed the sample with epoxy glue and fixed its thickness. The air trapped at the corners and edges is more sensitive to pressure changes and can induce a small flow in the colloid when a mechanical force is applied. We manually used an objective attached with a spring to press the coverslip and apply the pressure. The measured flow rates in the fields of view were very small, and thus their fluctuations were relatively large (Supplementary Fig. 5). Nevertheless, the fluctuations were much slower than the rapid martensitic processes, which typically took <30 s (Figs 1, 2, 3). Although different experimental trials produced different flow rates with some fluctuations, the kinetic pathways in each regime of Fig. 5 of the main text are robust to the average flow rate. We recently applied well-controlled flows using a microfluidic device (Supplementary Fig. 10 and Supplementary Note 2), and also observed the same behaviours: intermediate liquid without flow and no intermediate liquid at the small flow.

The typical grain size of the polycrystals ranged from 10 to 300 μm. A 0.4 μl colloidal suspension usually assembled into four layers at the centre and five or six layers near the edges. Therefore, the wall bending was small and the thickness was rather uniform over a field of view of ∼100 μm. The glass surfaces were rigorously cleaned so that particles would not stick to the walls. The refractive indexes of NIPA spheres and water are very close because more than 90% of the microgel was water. Consequently, bulk layers can be seen clearly in images even under the bright field when the spheres were packed into a crystal^22^. However, images became blurry when the spheres formed a liquid. We typically observed a surface layer because liquid-like particles produced a clearer image. Before the experiment, we used the temperature controller to cycle the temperature slightly below the transition point to anneal defects away and release possible pressure during sample preparation. The sample temperature was adjusted using the temperature controller (Bioptechs) on the microscope with a 0.1 °C resolution. To avoid the s–s transition from the pre-existing *n*✓–(*n*−1)Δ interfaces (Supplementary Movie 1 of ref. 29), we locally superheated the interior of an *n*✓ crystalline domain using a beam of light from a mercury lamp; the ambient temperature remains below the s–s transition point (Supplementary Fig. 4). This optical heating technique has been used in our previous crystal melting experiment^23^. A paraffin film was placed in the light path to achieve uniform optical heating. We measured the heating effect from *δT*=*T*_m_−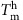, where 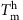 and *T*_m_ are the melting temperatures at a grain boundary with and without optical heating, respectively. *δT* can be controlled by adjusting the light intensity and dye concentration, and was set to 1.6 °C. The heating profile shown in Supplementary Fig. 4b was measured from an aqueous solution of yellow fluorescein (0.01% by weight) in a cell 5 μm thick. The brightness of the fluorescent solution was proportional to the light intensity and the heating effect^60^. The light from the mercury lamp was focused by the objective, hence the strongest heating effect is at the focal plane. From the melting of the 3D NIPA colloidal crystals, we found that the temperature changed by <0.1° in ±10 layers along the *z* direction^23^, hence the temperature was uniform enough in the five-layer-thick sample. Supplementary Fig. 2c shows that the heating effect (*δT*=1.6 °C) can quickly stabilize in 3 s after the light was turned on. Therefore, the observed nucleation processes were in steady states.

### Data analysis

Since the diameter and volume fraction cannot be determined exactly for soft spheres, we use the Lindemann parameter *L* to characterize the volume fraction *φ*. The results can be directly compared with *L* in atomic crystals. To avoid the slow divergence of mean-square displacement (MSD) in 2D, a modified dynamic Lindemann parameter *L* based on the local coordinates of neighbouring particles is often used^61^:


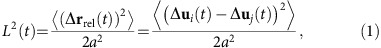


where *a* is the lattice constant measured from the position of the first peak of the radial distribution function *g*(*r*), Δ**r**_rel_ is the relative neighbour–neighbour displacement, Δ**u**_*i*_ is the displacement of particle *i*, particles *i* and *j* are nearest neighbours, and 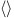 is the ensemble average. The factor 1/2 arises from the fact that the Lindemann parameter describes the displacement relative to the equilibrium position, while the dynamic MSD 〈(Δ**r**_rel_(*t*))^2^〉 describes the displacement relative to a previous time. *L* is measured from −2 to +2 s around each frame and is colour-coded in Figs 1, 2, 4, 8 and 9, Supplementary Fig. 7 and in Supplementary Movies 1,3,5,6,8 and 9 according to the colour bar shown above Fig. 1p of the main text. The dynamic MSD reaches a plateau due to the caging of neighbouring particles in <1 s (Supplementary Fig. 6), and thus 4 s trajectories are long enough for calculating *L*. A dislocation can enhance the *L* value of its four layers of neighbours^22^, which is approximately the distance between the newly formed nucleus and the pre-existing dislocation (Fig. 2b of the main text). 2D local orientational orders 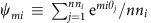 (ref. 59). 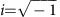. *m*=4 and 6 correspond to four- and six-fold symmetries, respectively. *θ*_*j*_ is the orientational angle of the bond between particle *i* and its nearest neighbour *j*. *nn*_*i*_ is the number of nearest neighbours for particle *i*. The nearest neighbours are identified from the Delaunay triangulations, which yield 〈*nn*〉=6 for any distribution of particles in 2D. Hence it is ideal for triangular lattices, but not for square lattices whose 〈*nn*〉=4. Therefore, the nearest neighbours for square lattices are further confined to those particles within a distance of <1.2*a*, which is the midpoint between the lattice constant *a* and the distance to the second nearest neighbour 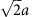 (ref. 59). A crystalline bond is defined as 
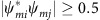
 (ref. 62). A ✓- or Δ-crystalline particle is defined as a particle with more than two four-fold crystalline bonds or more than three six-fold crystalline bonds, respectively. Other particles are defined as liquid. Liquid particles can be accurately identified since they must satisfy both the bond-orientational order criterion and the Lindemann parameter criterion, and are not very sensitive to threshold changes. Moreover, we confirmed the identified liquid-like particles from the videos: liquid-like particles swapped positions, but solid-like particles did not.

### Data availability

The data that support the findings of this study are available on request from the first author Y.P. or the corresponding author Y.H.

## Additional information

**How to cite this article:** Peng, Y. *et al*. Diffusive and martensitic nucleation kinetics in solid-solid transitions of colloidal crystals. *Nat. Commun.* **8,** 14978 doi: 10.1038/ncomms14978 (2017).

**Publisher's note**: Springer Nature remains neutral with regard to jurisdictional claims in published maps and institutional affiliations.

## Supplementary Material

Supplementary Information Supplementary Figures, Supplementary Notes and Supplementary References

Supplementary Movie 1 The precursor, nucleation and growth processes in a locally defect-free region shown in Figure 1. The left part is the raw video and the right part is the same video but with the colour-coded Lindemann parameters. 40 × real time.

Supplementary Movie 2 The formation and motion of dislocation pairs and the martensitic nucleation shown in Figs. 1a-l. Real time.

Supplementary Movie 3 The precursor, nucleation and growth processes near a dislocation shown in Figs. 2a-g. The left part is the raw video and the right part is the same video but with the colour-coded Lindemann parameters. 40 × real time.

Supplementary Movie 4 The formation and motion of dislocation pairs and the martensitic nucleation shown in Figs. 2a-d. Real time.

Supplementary Movie 5 The growth of a nucleus on a grain boundary. The upper facet is incoherent whereas the lower one is coherent. The left part is the raw video and the right part is the same video but with the colour-coded Lindemann parameters. 40 × real time.

Supplementary Movie 6 The incomplete nucleation process shown in Fig. 4. The left part is the raw video and the right part is the same video but with the colour-coded Lindemann parameters. 40 × real time.

Supplementary Movie 7 The strain fields of γ (left) and D^2^ (right) in during the nucleation shown in Fig. 1 and 7. The colour-coded γ and D^2^ are same as those in Figs. 6 and 7. 5 × real time.

Supplementary Movie 8 The nucleation and growth processes near a grain boundary shown in Fig. 8. The left part is the raw video and the right part is the same video but with the colour-coded Lindemann parameters. 40 × real time.

Supplementary Movie 9 The nucleation and growth processes near a triple junction with a negligible flow (2 nm s^-1^) shown in Fig. 9. The left part is the raw video and the right part is the same video but with the colour-coded Lindemann parameters. 40 × real time.

## Figures and Tables

**Table 1:** Nucleation kinetics in the *n*✓→(*n*−1)Δ transition under different flow rates and near different defects.

**Location**	**Inside domain, at grain boundary or asymmetric triple junction**	**Inside domain (that is, defect-free region or near dislocation)**	**Grain boundary**	**Symmetric triple junction**
Flow rate	*ν*≲10 nm^−1^ s	10≲ν≲100 nm s^−1^	10≲ν nm s^−1^	≲100 nm s^−1^
Precursor	Particle swapping	Dislocation pairs	Particle swapping	Particle swapping
Pathway	✓→liquid→Δ	✓→Δ	✓→Δ	✓→Δ
Particle motion	diffusive	Martensitic followed by diffusive	Diffusive	Diffusive
Lattice orientation	Mainly random	[10]_Δ_ || [11]_✓_	[10]_Δ_ || [10]_✓_	[10]_Δ_ || [10]_✓_
Shape evolution	Circle→polygon	Ellipse→parallelogram	Polygon	Equilateral triangle
Facet structure	Mostly or all incoherent	Incoherent	One or two coherent	All coherent
Example	Supplementary Fig. 7 and ref. 29	Figs 1, 2, 3	Fig. 8	Fig. 9

The two-step pathway in the first column was previously reported in ref. 29. Besides these four pathways, incomplete martensitic nucleation occurs within crystalline domains at high *φ* and high *ν* (Figs 4 and 5a).
