# Supplementary material for: Diffusive and martensitic nucleation kinetics in solid-solid transitions of colloidal crystals
Source: Nat Commun. 2017 May 15;8:14978. doi: 10.1038/ncomms14978 (PMC5440677; doi:10.1038/ncomms14978)
Supplement: Supplementary Information — Supplementary Figures, Supplementary Notes and Supplementary References [file ncomms14978-s1.pdf]

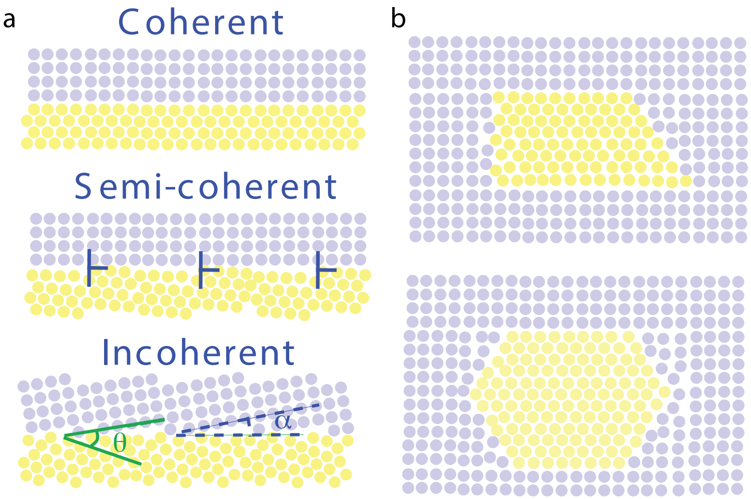

FIG. 1: (a) Structures of coherent, semi-coherent and incoherent interfaces. A coherent interface requires both an appropriate mismatch angle (labeled as θ) and an appropriate inclination angle (labeled as α) so that the two lattices would match perfectly at the interface. θ is the angle between the two lattices and α is the angle between the interface and one lattice orientation. The semi-coherent interface can be viewed as a chain of dislocations (labeled as $\perp$). (b) A $\triangle$-lattice nucleus embedded in a $\square$-lattice can have two coherent facets at most.

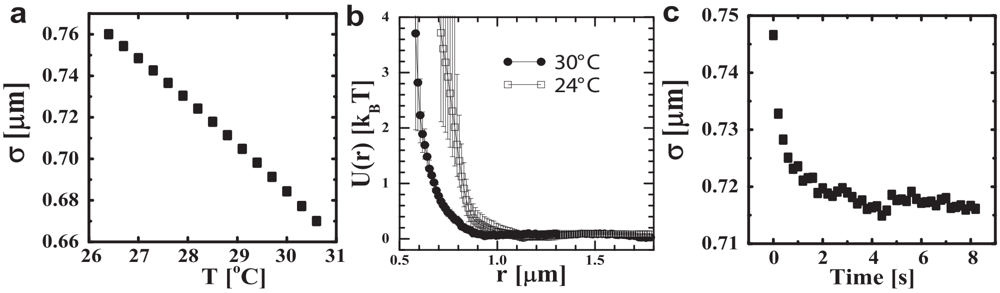

FIG. 2: (a) Mean effective diameter of well-isolated NIPA spheres stuck to a glass plate decreases with the temperature. (b) Pair potential $U(r)$ of NIPA microgel spheres derived from the measured radial distribution function $g(r)$ of a dilute monolayer of NIPA spheres strongly confined between two glass plates using the Ornstein-Zernike integral equation [1, 2]. (c) Mean diameter of particles stuck to a glass plate decreased in the first 3 s after switching on the heating light at $t = 0$ s.

Supplementary Figures

Supplementary Note 1 S-s transitions are usually first-order phase transitions accom-

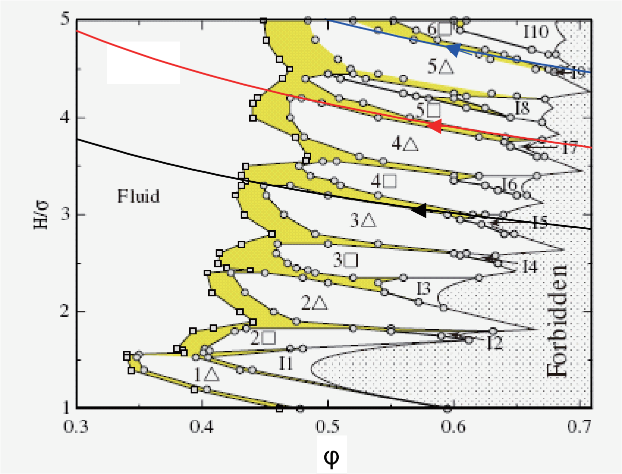

FIG. 3: Equilibrium phase diagram of hard spheres with diameter σ confined between two parallel hard walls with separation H (reproduced from the simulation work by A. Fortini and M. Dijkstra, J. Phys. Condens. Matter 18, L371 (2006) [3]). The white, yellow shaded and dotted regions indicate the stable one-phase region, the two-phase coexistence region, and the forbidden region, respectively. By varying σ, both the volume fraction ϕ and the ratio of H/σ changed, resulting in $n\square \rightarrow (n-1)\triangle$ transition paths for $n = 4$ (black curve), $n = 5$ (red curve), and $n = 6$ (blue curve) at different H.

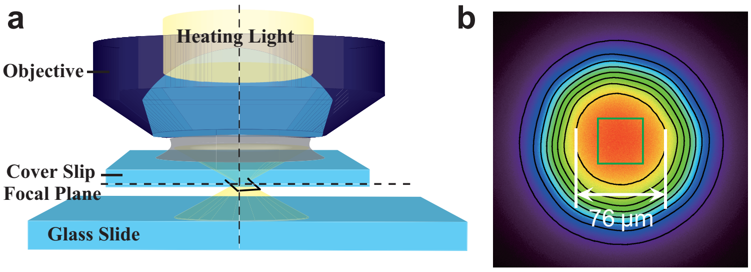

FIG. 4: (a) Schematic of local optical heating. (b) Measured temperature profile in the xy plane. The contour spacing is 0.2°C. The temperature difference in the $\pi(38\mu\text{m})^2$ area of the central circle is less than 0.2°C, which is larger than the field of view.

panied by an abrupt change in density. They are ubiquitous in nature and industry. For example, water has 17 crystalline structures [5], but only one or two liquid phases [6], hence it has many more types of s-s transitions than solid-liquid transitions. Some terminologies in s-s transitions were used with ambiguities. To clarify the related terminologies and con-

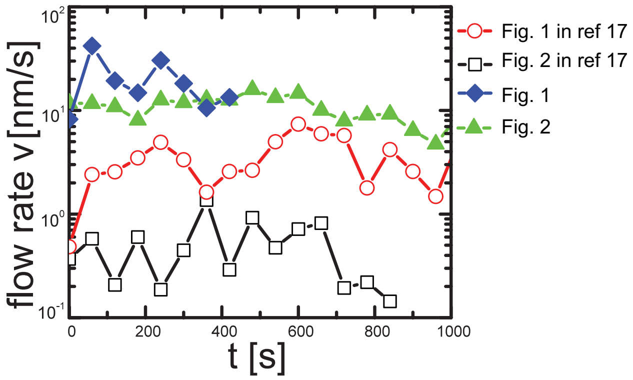

FIG. 5: Flow rate fluctuations in s-s transition samples. Flow rates were measured from the averaged drift speed over all particles in the region of interest. Open symbols represent the samples that underwent two-step 5□ → liquid → 4△ diffusive nucleation in ref. [4]. Solid symbols represent the samples that underwent one-step 5□ → 4△ martensitic nucleation followed by diffusive growth in Figs. 1, 2.

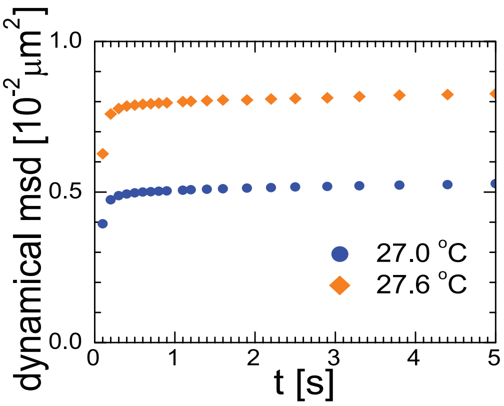

FIG. 6: Dynamic mean-square displacements in the xy plane averaged over all crystalline particles.

cepts in metallurgy which are seldom used in soft matter physics, we introduce them in the following. A more comprehensive categorization of s-s transition pathways [7] than that in the introduction would be (1) diffusive (i.e. civilian) transformations in which particles randomly diffuse from the parent phase to the product phase by nucleation or spinodal decomposition and (2) diffusionless (i.e. military) transformations which involve cooperative movement of many particles without long-range diffusion. Structure change involving inter-particle bond breaking is called reconstructive, otherwise it is displacive. In practice, however, many phase transitions with some of bonds being broken were also assorted as

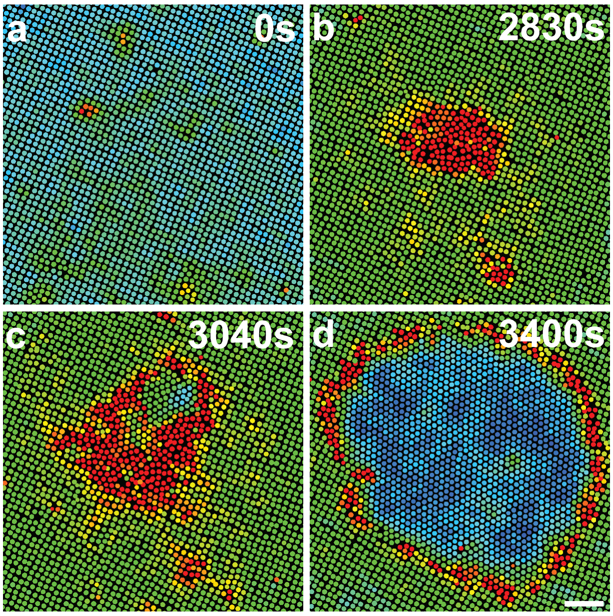

FIG. 7: Example of the two-step diffusive nucleation of 5□ → liquid → 4△ (reproduced from ref. [4].) Colours represent different values of the Lindemann parameter L as in Fig. 1m-o. Scale bar: 5 μm. (a) Optical heating commenced at $t = 0$ s. (b) After incubation for approximately 2800 s, a post-critical liquid nucleus was developed and grew large in an irreversible manner. (c) At 3040 s, a △-lattice crystallite nucleated from the liquid nucleus. (d) At 3400 s, the whole nucleus became a △-lattice.

displacive and their bond-breaking mechanism at the microscopic scale remained poorly understood. Martensitic transformation is a major type of diffusionless transformation, whose kinetics and morphology are dominated by strain energy [8]. Note that diffusionless transformations are not always martensitic. For example, homogenous dilation of the whole crystal in an iso-structural s-s transition (e.g. ref. [9]) is a non-martensitic diffusionless transformation. Martensitic transformations follow the nucleation process, although the nucleus usually grows rapidly (e.g. close to the speed of sound). Displacive martensitic transformation, also called as weak martensitic transformation [10], exhibits reversible elastic deformation, while reconstructive martensitic nucleation involves irreversible plastic deformation concomitant with the breaking of inter-particle bonds. Sometimes, diffusionless, displacive and martensitic transformations were used interchangeably in literature, but they are distinct in this

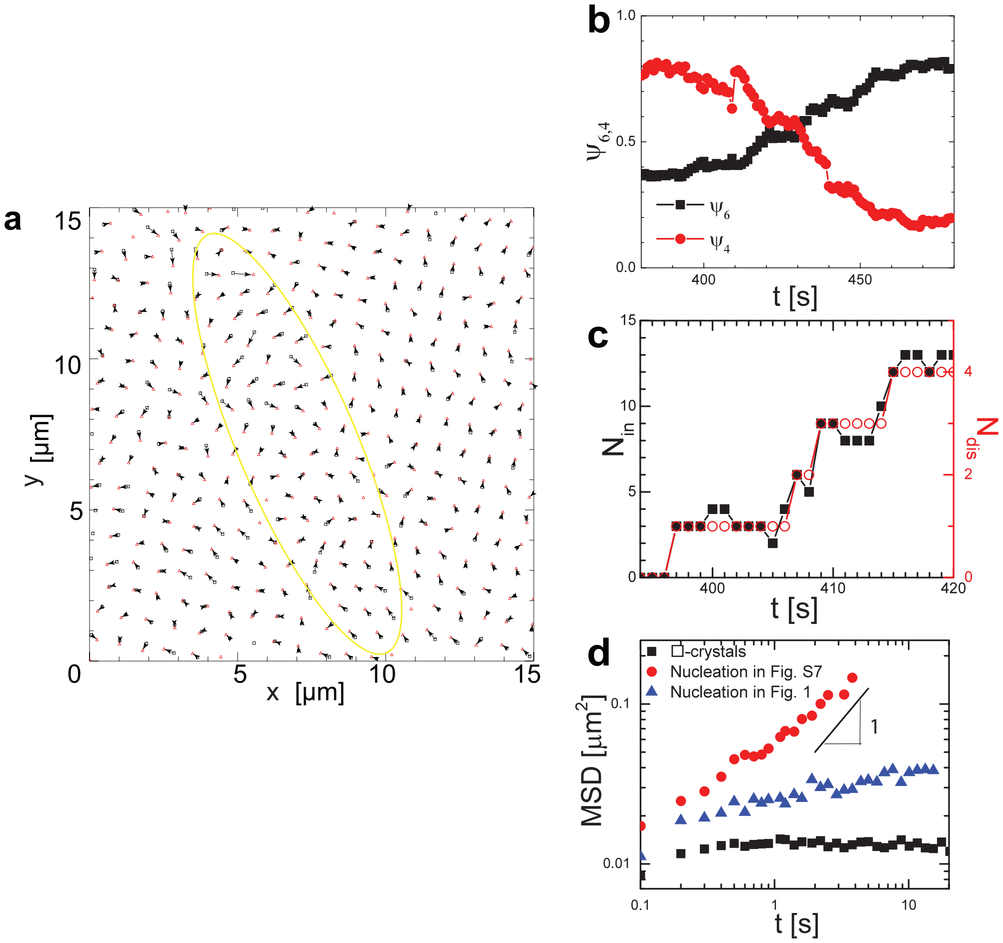

FIG. 8: Additional characterizations of the nucleation process in Fig. 1. (a) The collective displacements of particles from 415 s to 422 s. (b) The evolution of the average four- and six-fold orientational order parameters. (c) The evolution of the numbers of the inserted particles N_{in} and dislocation pairs N_{dis} . (d) The dynamical mean square displacements in the initial □ lattice, a liquid nucleus of two-step nucleation and the initial stage of a one-step nucleation. The long-time MSD for the liquid nucleus in a two-step nucleation confirms the diffusive behavior of the liquid. By contrast, the long-time MSD reaches a plateau for the nucleation in Fig. 1, indicating the absence of the liquid. (b, d) are measured only in the nucleus region.

paper. Note that diffusive transformations involve the breaking of inter-particle bonds and thus are reconstructive transitions. Anisotropic stress [11], rapid quenching [7] and small system size [12, 13] have been suggested to promote martensitic transformations, but their microscopic kinetics and mechanisms are unclear. Martensitic transformations occurs inside

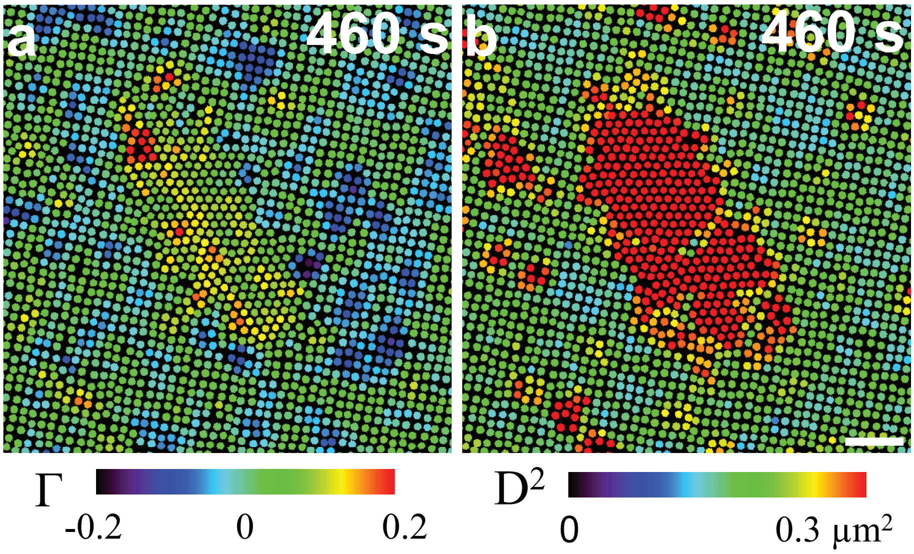

FIG. 9: The distributions of the shear strain (a) and non-affine parameter (b) in the later growth stage of the △-lattice nucleus in Fig. 1n. Around the △-lattice nucleus, shear strain was similar to that in the bulk □-lattice (a) rather than propagated at the growth front of the nucleus as shown in Fig. 7. The nonaffine zone grew at the same speed as the growth rate of nucleus (b). These two facts confirmed the reconstructive nature of the transformation in the nucleus growth stage.

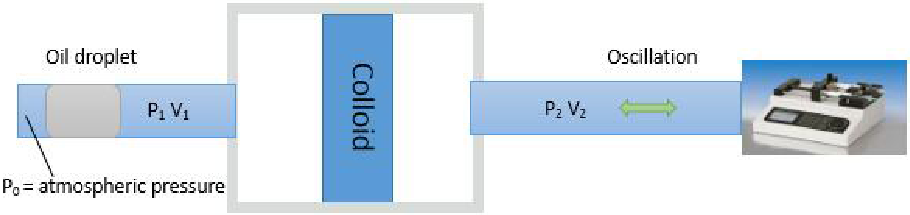

FIG. 10: The schematic of the pressure gradient measurement.

domains whereas diffusive transformations often occur on the domains boundaries [11]. The growth of a martensite nucleus is terminated when it encounters a grain boundary, the sample surface or another martensite [14]. In our experiment, particles in crystal phases were bound on their lattice sites without out-of-cage diffusions, however the nucleus growth is called diffusive because particles transformed from parent to the product phase, i.e. across the nucleus surface, via individual random diffusion instead of collective motions.

A grain boundary in a quasi-2D crystal can be characterized by one mismatch angle θ

between the two grain lattices and two inclination angles α of the grain boundary relative to the two lattices, as illustrated in Supplementary Fig. 1. $\theta = \alpha_1 + \alpha_2$, thus two of the three angles are independent. When the grain boundary moves, the two inclination angles would usually change, but the mismatch angle does not. The coherence of an interface depends on both the mismatch angle θ and inclination angle α. The lattice constants of our $\square$ and $\triangle$ lattices are almost the same, hence a coherent interface requires $\theta = \alpha = 0$. The interfacial energy is the lowest for coherent interfaces and the highest for incoherent interfaces. Some incoherent interfaces with special angles have lower energies, e.g. 45° for the incoherent facets III, VI in Fig. 1o, II and IV in Fig. 2e and III and IV in Fig. 8d.

Supplementary Note 2 The viscosity of the dense colloid is not available, hence it is difficult to directly estimate the pressure gradient responsible for the ∼10 nm/s flow. Therefore we measured the pressure gradient which drove the colloid at a high flow rate $v = 35$ μm/s and then calculated the pressure gradient at the low flow rate. The experimental setup is shown in Supplementary Fig. 10. The colloidal suspension was in the middle of a 18 mm × 18 mm × 0.1 mm glass cell whose two ends were connected to two volumes V_1 and V_2 filled with air. V_1 was sealed by an oil droplet (microscope immersion oil with density $\rho = 1.025$ g/mL) and V_2 was connected to a syringe pump (LongerPump LSP02-1B). The pump pushed the air at 1 Hz and oscillated the colloid with an amplitude of < 1 mm and the oil droplet with an amplitude of $A = 1$ cm. The colloid in the thin cell has a much higher friction than that of the oil droplet, hence its amplitude is much smaller. The oil droplet completely wetted the inner surface of the glass tube because it was covered by a thin film of oil. Since the oscillation is slow, we assume that the volume change of the air is an isothermal process rather than an adiabatic process. According to the idea gas law, the pressure change is $\mathrm{d}P = \mathrm{d}\left(\frac{Nk_\mathrm{B}T}{V}\right) = -\frac{Nk_\mathrm{B}T}{V}\frac{\mathrm{d}V}{V} = -P\frac{\mathrm{d}V}{V}$. The air between colloid and oil droplet has $V_1 = 0.555$ mL and $\Delta V_1 = 0.00785$ mL, thus $P_1 - P_0 = 1.43 \times 10^3$ Pa where the atmosphere pressure $P_0 = 1.01 \times 10^5$ Pa. $P_2 - P_0$ can be obtained similarly. The syringe pump changed the 60 mL air at 0.25 mL/s$\cdot \cos(2\pi\ \mathrm{Hz} \cdot t)$. Hence $P_2 - P_0 = 422$ Pa. Consequently, $P_2 - P_1 \simeq 1000$ Pa which drove the colloid moved at 35 μm/s. Since the colloidal crystal

drifted as a whole, it was more like a plug flow whose pressure gradient along the flow is $\mathrm{d}P/\mathrm{d}x \propto v^2/H$. Consequently, the colloid drifted at 10 nm/s in a 3 μm-thick cell was driven a pressure gradient roughly about 0.27 Pa/m.

---

[1] Polin, M., Grier, D. G. & Han, Y. Colloidal electrostatic interactions near a conducting surface. *Phys. Rev. E* **76**, 041406 (2007).

[2] Behrens, S. H. & Grier, D. G. Pair interaction of charged colloidal spheres near a charged wall. *Phys. Rev. E* **64**, 050401 (2001).

[3] Fortini, A. & Dijkstra, M. Phase behaviour of hard spheres confined between parallel hard plates: manipulation of colloidal crystal structures by confinement. *J. Phys.: Condens. Matter* **18**, L371 (2006).

[4] Peng, Y. *et al.* Two-step nucleation mechanism in solid–solid phase transitions. *Nature Mater.* **14**, 101–108 (2015).

[5] Falenty, A., Hansen, T. C. & Kuhs, W. F. Formation and properties of ice xvi obtained by emptying a type sii clathrate hydrate. *Nature* **516**, 231–233 (2014).

[6] Palmer, J. C. *et al.* Metastable liquid-liquid transition in a molecular model of water. *Nature* **510**, 385–388 (2014).

[7] Porter, D. A., Easterling, K. E. & Sherif, M. Y. *Phase transformations in Metals and Alloys* (CRC Press, 2008).

[8] Delaey, L. *Diffusionless Transformations* (Wiley Online Library, 2013).

[9] Bolhuis, P. & Frenkel, D. Prediction of an expanded-to-condensed transition in colloidal crystals. *Phys. Rev. Lett.* **72**, 2211–2214 (1994).

[10] Bhattacharya, K., Conti, S., Zanzotto, G. & Zimmer, J. ,. *Nature* **428**, 55–59 (2004).

[11] Burnley, P. C. & Green, H. W. Stress dependence of the mechanism of the olivine-spinel transformation. *Nature* **338**, 753 (1989).

[12] Scandolo, S., Bernasconi, M., Chiarotti, G. L., Focher, P. & Tosatti, E. Pressure-induced transformation path of graphite to diamond. *Phys. Rev. Lett.* **74**, 4015–4018 (1995).

[13] Khaliullin, R. Z., Eshet, H., Kühne, T. D., Behler, J. & Parrinello, M. Nucleation mechanism for the direct graphite-to-diamond phase transition. *Nature Mater.* **10**, 693–697 (2011).

[14] Fultz, B. *Phase Transitions in Materials* (Cambridge University Press, 2014).
